# Serum Adiponectin Level as a Predictor of Subclinical Cushing's Syndrome in Patients with Adrenal Incidentaloma

**DOI:** 10.1155/2016/8519362

**Published:** 2016-08-30

**Authors:** Asli Dogruk Unal, Semra Ayturk, Derya Aldemir, Neslihan Bascil Tutuncu

**Affiliations:** ^1^Memorial Atasehir Hospital, Department of Endocrinology and Metabolism, Istanbul, Turkey; ^2^Trakya University Hospital, Department of Endocrinology and Metabolism, Edirne, Turkey; ^3^Baskent University Hospital, Department of Biochemistry, Ankara, Turkey; ^4^Baskent University Hospital, Department of Endocrinology and Metabolism, Ankara, Turkey

## Abstract

Subclinical Cushing's syndrome (SCS) is a condition of slight but chronic cortisol excess in patients with adrenal incidentaloma (AI) without typical signs and symptoms of Cushing's syndrome. Adiponectin has potent roles in modulating energy balance and metabolic homeostasis and acts in opposition to glucocorticoids. This study aimed to evaluate adiponectin level in SCS and nonfunctional AI (NAI) patients and its relation with metabolic parameters. Patients with AI (*n* = 40) and metabolically healthy controls (*n* = 30) were included. In AI patients and controls, detailed medical history assessment, physical examinations, anthropometric measurements, and laboratory measurements were performed. Age, body mass index, waist circumference, and lipid profiles were significantly higher and waist-to-hip ratio and adiponectin level were significantly lower in the AI patients than in the controls. The midnight cortisol and urinary free cortisol levels were significantly higher in the SCS patients (*n* = 8) than in the NAI patients (*n* = 32). Adiponectin level of the SCS group was significantly lower than those of the NAI and control groups. The sensitivity and specificity for an adiponectin level of ≤13.00 ng/mL in predicting the presence of SCS were 87.5% and 77.4%, respectively. In conclusion, adiponectin is valuable in predicting the presence of SCS in AI patients.

## 1. Introduction

Adrenal incidentaloma (AI) is an adrenal mass that is typically discovered serendipitously during radiologic examinations performed for unrelated reasons [1]. AI is observed in approximately 5% of all abdominal computed tomography (CT) scans and its prevalence in the general population has been estimated as 3%–7% [2]. The prevalence of AI increases with age; the likelihood of encountering an AI on CT is 10% in patients aged 70 years and older [3].

Most of the AIs are benign adenomas and do not exhibit hyperfunction [2]. Nevertheless, pheochromocytoma, aldosteronism, or overt cortisol excess due to hyperfunction can be observed in patients with AI [4]. Autonomous cortisol (a glucocorticoid hormone) secretion in patients with features similar to Cushing's syndrome (CS) such as obesity, hypertension, glucose intolerance, and osteoporosis but without typical signs or symptoms of hypercortisolism is defined as subclinical CS (SCS) [4–6]. The clinical presentation of CS varies according to the extent and duration of cortisol hypersecretion; however, proximal muscle wasting, wide purple striae, and easy bruising are the unique signs of CS. Specifically, patients with hypercortisolism usually present with central obesity, a round and erythematous face, supraclavicular fat pads, cervical fat accumulation called buffalo hump, thinning of the skin, and proximal muscle weakness [7–10]. In patients with SCS, slight but chronic cortisol release has been associated with increased cardiovascular risks and metabolic syndrome [11, 12]. SCS diagnosis is established via abdominal CT scan in patients having an incidental adrenal mass, without the above-mentioned typical physical features of CS, and exhibiting endogenous hypercortisolism in hormonal tests. Nevertheless, there are no universally accepted hormonal criteria for SCS diagnosis [6, 13–16]. A cortisol value of >5 *μ*g/dL after 1-mg dexamethasone suppression test (1-mg DST) is accepted as a sufficient criterion to diagnose SCS [15–17]. Patients with cortisol values between 1.8 *μ*g/dL and 5 *μ*g/dL in this test should also have at least one other criterion: lack of circadian cortisol secretion rhythm (late evening to morning serum cortisol % ratio exceeded 50%), decreased morning adrenocorticotropic hormone (ACTH) concentration (≤10 pg/mL), and/or increased 24-hour urinary free cortisol excretion [15–17].

Adiponectin, a peptide with 244 amino acids, is a very important hormone that is secreted primarily from white adipose tissue. Adiponectin is involved in metabolic processes and negatively correlates with obesity [18]. Glucocorticoids and adiponectin play opposing roles in regulating energy metabolism, where glucocorticoids activate catabolic processes and insulin resistance. Adiponectin acts primarily as an insulin sensitizer [18, 19]. There is growing evidence that adiponectin has protective effects against type 2 diabetes mellitus [20]. Additionally, with respect to immune and inflammatory processes, both glucocorticoids and adiponectin have anti-inflammatory effects [21, 22]. Thus, there is an increasing interest in the clinical application of adiponectin on variety of diseases such as dyslipidemia, metabolic syndrome, hypertension, and cancer (breast, colon, and prostate cancers) [23]. Drugs enhancing endogenous adiponectin production or using recombinant adiponectin attract attention as the potential treatment targets [24].

It is known that the metabolic abnormalities of SCS remain elusive [25]. Surgical removal of autonomously cortisol-secreting adenoma has beneficial effects on blood pressure and insulin sensitivity [26, 27]; however, there are still insufficient data to support routine adrenalectomy in the clinical setting [28]. In a study, the patients with CS were observed to have low adiponectin levels; however, obesity was reported to mask the relationship between adiponectin and cortisol [29]. The present study aimed to evaluate adiponectin levels in patients with SCS and nonfunctional AI (NAI) and its relation with metabolic parameters.

## 2. Materials and Methods

### 2.1. Subjects

Patients (*n* = 40) referred to the Endocrinology and Metabolic Disease Outpatient Clinic for incidentally discovered adrenal masses were included in the present study. The control group included metabolically healthy subjects (*n* = 30) without adrenal adenomas or hyperplasia. Patients with type 1 diabetes mellitus, uncontrolled hypertension, chronic renal failure, chronic hepatic disease, depression, or alcoholism or those who were on medication that could influence insulin, cortisol, and dexamethasone metabolisms or cortisol secretion were excluded from the study. All the patients diagnosed with type 2 diabetes mellitus were newly diagnosed patients and not on any antidiabetic treatment. The patients included in the present study were not using statins for about 12 weeks.

In AI patients and controls, detailed medical history assessment, physical examinations, and anthropometric measurements were performed. Blood samples were obtained at 08:00 a.m. for the measurements of basal cortisol, ACTH, dehydroepiandrosterone sulfate (DHEAS), fasting blood glucose (FBG), and insulin levels as well as lipid profiles and adiponectin levels. Additionally, 24-hour urinary cortisol excretion and midnight cortisol level were measured and 1-mg DST was performed in the SCS and NAI patients.

The study was approved by the Baskent University Institutional Review Board and Ethics Committee (Project number KA07/69) (Ethics Committee for Baskent University Hospitals) and conducted in accordance with the Declaration of Helsinki II. All participants provided written informed consent before participating in the study.

### 2.2. Measurements

All incidentalomas discovered by abdominal ultrasound or magnetic resonance imaging (MRI) were evaluated by CT. On CT, all adrenal masses were homogenous, hypodense, and well shaped which were the features consistent with the diagnosis of adrenocortical adenoma. No patient had evidence of metastatic disease. In each patient, three diameters of the adrenal mass were measured and the largest diameter was recorded.

The 24-hour urinary metanephrines and the aldosterone-to-renin ratio were within normal ranges in AI patients, thus excluding pheochromocytoma and aldosteronoma. All AI patients with hypertension initiated verapamil 6 weeks before functional evaluation. None of the patients had overt signs or symptoms of CS. The diagnosis of SCS was based on the presence of at least one of the following in addition to cortisol levels greater than 1.8 *μ*g/dL after 1-mg DST [13]: (1) urinary free cortisol (UFC) levels of >300 *μ*g/day in two of the three consecutive collections per 24-hour period, (2) ACTH levels of <10 pg/mL (<2.2 pmol/L), and (3) low serum DHEAS levels.

The body mass index (BMI) was calculated as weight in kilograms divided by height in meters square (kg/m^2^). The waist-to-hip ratio was calculated as the ratio between the smallest circumference of the torso and the maximum circumference of the hips over the buttocks. Body composition was measured by bioelectrical impedance analysis using the TANITA TBF-300 Body Composition Analyzer (Tanita Corp., Tokyo, Japan). Anthropometric measurements were obtained using standardized equipment and following the recommendations of the International Biological Program.

Serum cortisol, UFC, and DHEAS levels were determined using commercial radioimmunoassay (RIA) kits (Immulite 2000; Diagnostic Products Corp., Los Angeles, CA, USA). ACTH levels were measured using an immunoradiometric assay (IRMA) (Immulite 2000). The 24-hour urine-fractionated metanephrines and vanillylmandelic acid (VMA) were measured using high-pressure liquid chromatography, in which the container was acidified with 10 mL to 25 mL of 6 N HCl for the preservation of the metanephrines. Before urine collections for VMA testing, patients were asked to avoid salicylates, caffeine, tea, chocolate, bananas, and vanilla for 72 hours. Plasma aldosterone concentrations (PAC) were measured by RIA and quantitative determination of plasma renin activity (PRA) was done by the enzyme-linked immunosorbent assay (ELISA). The blood sample was collected in a tube containing ethylenediaminetetraacetic acid. The lower limit of the assay for the detection of PRA was 0.1 ng/mL/h. The cut-off value for PAC (in ng/dL)/PRA (in ng/mL/h) ratio was accepted as 20 for hyperaldosteronism.

For the measurement of FBG, an enzymatic colorimetric method with glucose oxidase was used. Lipid profiles, including triglycerides, high-density lipoprotein cholesterol (HDL-C), and low-density lipoprotein cholesterol (LDL-C), were assessed using commercially available assay kits (Roche Diagnostics, GmbH Roche Molecular Biochemicals, Mannheim, Germany). Insulin levels were measured using the Immulite 2000 immunoassay analyzer (Euro/DPC, Llanberis, UK). The HOMA-IR was estimated according to the following formula [30]: HOMA-IR = [FBG (mmol/L) × Fasting insulin level (mU/L)]/22.5.

Adiponectin levels were measured in duplicate using a commercially available ELISA kit (Linco Research, St. Charles, MO, USA). The sensitivity of the assay was 0.78 ng/mL. The intra-assay and interassay coefficients of variation were less than 7.4% and 8.4%, respectively.

### 2.3. Statistical Analyses

Data analyses were performed using the PASW Statistics for Windows, version 18.0 (SPSS Inc., Chicago, IL, USA). Descriptive statistics were expressed as number and percentage for categorical variables and as mean, standard deviation, median, 25th percentile (*Q*1), 75th percentile (*Q*3), and minimum and maximum for numerical variables. For the comparisons of independent numerical variables, when normal distribution assumption was not met, Mann–Whitney *U* test was used for two group comparisons and Kruskal-Wallis test was used for multiple group comparisons. For subgroup analyses, Mann–Whitney *U* test was performed with the Bonferroni correction. Chi-square test was used for between-group comparisons of categorical variables. In order to determine the relationship between adiponectin level and numerical variables, Spearman's rho test was used when normal distribution assumption was not met. The receiver operating characteristics (ROC) analysis was used to determine the value of adiponectin level in predicting the presence of AI and SCS. The statistical significance was evaluated at a type 1 error of <5%. The independent effects of age, gender, waist circumference, hip circumference, waist-to-hip ratio, body fat percentage, fat-free mass (FFM), insulin, HOMA-IR, LDL-C, HDL-C, triglyceride, and study group variables, which yielded a *p* value of <0.200, on adiponectin were investigated by performing multivariate linear regression analysis. Thereafter, a model including age, gender, hip circumference, HOMA-IR, LDL-C, and study group variables was developed. For linear regression analysis, a logarithmic transformation was performed for adiponectin levels.

## 3. Results

The present study included 40 AI patients (24 females and 16 males) with a mean age of 60.5 ± 8.2 years (range, 45–76 years). The control group included 30 subjects (22 females and 8 males) with a mean age of 26.0 ± 4.4 years.

The clinical, demographic, and metabolic characteristics of the AI patients and controls are shown in Table 1. Age, BMI, waist circumference, and levels of total cholesterol, LDL-C, HDL-C, and triglyceride were significantly higher and waist-to-hip ratio and adiponectin level were significantly lower in the AI patients than in the controls.

Among the AI patients, 8 (20%) were diagnosed with SCS and the remaining patients (80%) were classified as NAI. The hormonal profiles and the diameters of adrenal adenomas in the SCS and NAI patients are shown in Table 2. The midnight cortisol and UFC levels were significantly higher in the SCS patients than in the NAI patients. Of the 8 patients with SCS, 5 had high midnight cortisol levels, 3 had high urinary cortisol excretion, and 4 had low DHEAS levels. The patients with ACTH levels >10 pg/mL were evaluated using pituitary MRI and no pituitary adenomas were detected.

Comparison of the SCS, NAI, and control groups revealed that age, BMI, waist circumference, insulin levels, HOMA-IR, lipid profiles, and adiponectin levels were significantly differed among the groups (Table 3).

**Figure 1:**
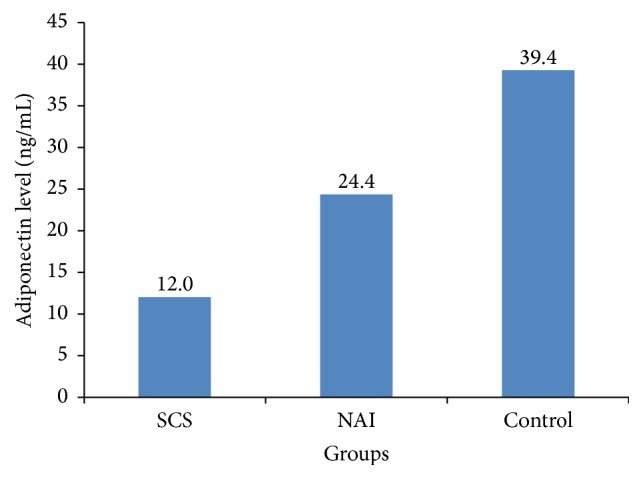
Adiponectin levels in the study groups. SCS, subclinical Cushing's syndrome; NAI, nonfunctional adrenal incidentaloma.

Two group comparisons revealed that only adiponectin level was significantly lower in the SCS group than in the NAI group (*p* = 0.007); there was no significant difference between the two groups in terms of other parameters. Age (*p* = 0.001), insulin level (*p* = 0.002), and HOMA-IR (*p* = 0.003) were significantly higher and adiponectin level (*p* < 0.001) was significantly lower in the SCS group than in the control group. Age (*p* < 0.001), BMI (*p* = 0.003), waist circumference (*p* < 0.001), and the levels of total cholesterol (*p* < 0.001), LDL-C (*p* = 0.006), HDL-C (*p* = 0.010), and triglyceride (*p* = 0.001) were significantly higher in the NAI group than in the control group. Insulin levels, HOMA-IR, and adiponectin levels were similar in the NAI and control groups. As a result, the adiponectin level of the SCS group was significantly lower than those of both NAI and control groups (Figure 1).

Adiponectin level was significantly and negatively associated with insulin level, HOMA-IR, triglyceride level, FFM, and midnight cortisol level and it was significantly and positively associated with body fat percentage and HDL-C and ACTH levels (Table 4). The linear regression analysis conducted to determine the risk factors affecting adiponectin level in a model including age, male gender, HOMA-IR, LDL-C, waist circumference, and presence of SCS revealed that age was a significant increasing factor, whereas male gender, HOMA-IR, LDL-C, waist circumference, and presence of SCS were significant decreasing factors (Table 5).

**Figure 2:**
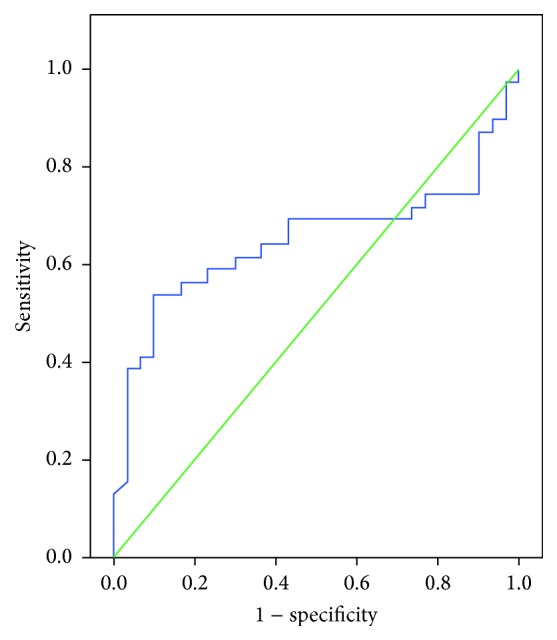
Receiver operating characteristics (ROC) curve for adiponectin level in predicting the presence of adrenal incidentaloma (AI).

Evaluation of the ROC analysis revealed that adiponectin level had a predictive value in determining the presence of AI (area under the curve (AUC): 0.65, confidence interval (CI): 0.52–0.78, and *p* = 0.034). The sensitivity, specificity, positive predictive value, and negative predictive value for an adiponectin level of ≤22.07 ng/mL in predicting the presence of AI were 54.6%, 83.3%, 81.5%, and 59.5%, respectively (Figure 2).

**Figure 3:**
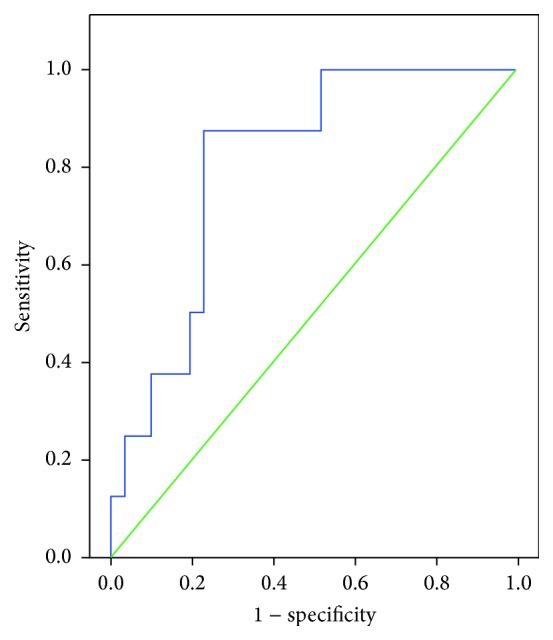
Receiver operating characteristics (ROC) curve for adiponectin level in predicting the presence of subclinical Cushing's syndrome (SCS).

Evaluation of the ROC analysis also revealed that adiponectin level had a predictive value in determining the presence of SCS (AUC: 0.81, CI: 0.67–0.96, and *p* = 0.007). The sensitivity, specificity, positive predictive value, and negative predictive value for an adiponectin level of ≤13.00 ng/mL in predicting the presence of SCS were 87.5%, 77.4%, 50.0%, and 96.0%, respectively (Figure 3).

## 4. Discussion

Histologically, AIs are mostly adrenocortical adenomas. Of these cases, approximately 1.7% develop hyperfunction and the risk is higher in the lesions greater than 3 cm [31]. Cortisol hypersecretion is the most frequently encountered hyperfunction and progresses subclinically in approximately two-thirds of the cases [31]. In various studies, the frequency of SCS has been reported as 5%–47% in AI patients depending on the study protocol and diagnostic criteria [32]. In the present study, in selected AI patients with benign adrenal lesions and without evidence of hyperaldosteronism or pheochromocytoma, the frequency of SCS was 20%. The median adenoma diameter in the patients with SCS was 3.05 cm. The midnight cortisol level (median 9.15 versus 5.10 *μ*g/dL, *p* = 0.004) and the UFC level (median 249 versus 170 *μ*g/24 h, *p* = 0.007) were significantly higher in the SCS patients than in the NAI patients.

Chronic release of glucocorticoids is associated with cardiovascular risk factors including abdominal obesity, insulin resistance, impaired glucose tolerance, diabetes mellitus, atherosclerosis, systemic hypertension, and dyslipidemia [33–37]. As these factors strongly affect morbidity and mortality, awareness of SCS is of importance. Conflicting results regarding decreased insulin sensitivity in NAI patients compared with SCS patients have been reported [12, 35, 38]. In the present study, the HOMA-IR and insulin level of the SCS patients were significantly higher than the controls. The lipid levels, BMI, and waist circumference were significantly higher in the NAI patients than in the controls. These parameters were also higher in the SCS group than in the controls; however, the difference did not reach statistical significance. This finding might be resulting from the small number of the patients in the SCS group. Elevated midnight cortisol concentration (>5 *μ*g/dL) has been reported as a marker of increased cardiovascular risk in AI patients [39]. In the present study, while the midnight cortisol level was 5.10 *μ*g/dL in the NAI patients, it was 9.15 *μ*g/dL in the SCS patients and the difference was statistically significant.

Adipose tissue secretes a number of cytokines and bioactive compounds, which are called adipokines, leading to metabolic alterations and cardiovascular morbidity [40]. Adiponectin is an adipokine that is primarily produced by visceral fat and circulates in the blood in different forms of varying molecular weights [41]. A low level of adiponectin is a common feature of obesity and is associated with insulin resistance [42]. Adiponectin also plays an important role in the development of cardiovascular risk by affecting proinflammatory molecules [43]. Although there are studies investigating the association between adiponectin and glucocorticoids, the results are inconclusive due to different transcription factors, signaling cascades, and hormones that regulate adiponectin expression [44]. Glucocorticoids regulate many different genes, signaling cascades, and transcription factors, some of which can regulate adiponectin expression [45, 46]. Although increased cardiovascular risk factors are observed in SCS patients, no standard treatment strategies exist. Therefore, we evaluated adiponectin levels in SCS patients. To the best of our knowledge, this is the first study to evaluate adiponectin levels in the SCS patients. As we hypothesized, we found the adiponectin levels to be lower in the SCS patients. The adiponectin levels of the SCS patients (12.0 ng/mL) were significantly lower than those of the NAI patients (24.4 ng/mL) and controls (39.4 ng/mL). In the present study, adiponectin level was found to be positively correlated with ACTH and HDL-C and negatively correlated with insulin, HOMA-IR, triglyceride, FFM, and midnight cortisol levels in all participants included in the present study. In a study, the adiponectin level was reported to be higher in the NAI patients than in the healthy controls and not related to the visceral obesity and it was hypothesized that adiponectin might be produced from the adrenal gland [47]. In the present study, adiponectin level was lowest in the SCS group and highest in the healthy control group, with a statistically significant difference between the SCS group and controls. Accordingly, we did not think that adiponectin was originated from the adrenal glands. In addition, we did not find a correlation between altered hypophysis-adrenal axis and adiponectin levels. In the study by Wallia et al. [48], adiponectin level was significantly increased with mifepristone throughout the course of treatment in the patients with CS. This finding also supports our result of low adiponectin level in the SCS patients.

In the present study, a ROC analysis was performed to determine the value of adiponectin in predicting the presence of AI and SCS. Accordingly, we found that the sensitivity, specificity, positive predictive value, and negative predictive value for an adiponectin level of ≤22.07 ng/mL in predicting the presence of AI were found as 54.6%, 83.3%, 81.5%, and 59.5%, respectively. The sensitivity, specificity, positive predictive value, and negative predictive value for an adiponectin level of ≤13.00 ng/mL in predicting the presence of SCS were found as 87.5%, 77.4%, 50.0%, and 96.0%, respectively.

## 5. Conclusions

To the best of our knowledge, this is the first study demonstrating low levels of adiponectin in patients with SCS. Presence of SCS should be considered in case of an adiponectin level of ≤13.00 ng/mL in AI patients. Low adiponectin levels in SCS patients may be important in treatment decision due to the known relation between adiponectin and cardiovascular events. In order to increase the evidences on this subject, further prospective follow-up studies with larger number of subjects are needed.

## Figures and Tables

**Table 1:** Demographic, clinical, and metabolic characteristics of the adrenal incidentaloma patients and control subjects.

	Patients with AI (*n* = 40)	Control subjects (*n* = 30)	*p*
	*n* (%)	*n* (%)	
Gender			
Female	24 (60.0)	22 (73.3)	0.245
Male	16 (40.0)	8 (26.7)	

	Median (*Q*1–*Q*3)	Median (*Q*1–*Q*3)	

Age, years	60 (55.5–65.5)	32 (24–44)	**<0.001**
Body mass index, kg/m^2^	29.1 (25.8–31.7)	25.8 (22.8–28.3)	**0.004**
Waist circumference, cm	97 (90.5–104)	85 (83–90)	**<0.001**
Waist-to-hip ratio	0.90 (0.85–0.94)	0.93 (0.92–0.95)	**0.049**
Body fat percentage, %	33.9 (24.6–41.9)	29.1 (26.0–33.0)	0.097
Fat-free mass, kg	48.6 (44.9–55.9)	44.8 (41.1–56.8)	0.151
FBG, mg/dL	93.5 (88–110)	91 (88–97)	0.147
Insulin, *μ*IU/mL	8.8 (5.6–11.2)	6.6 (4.8–8.1)	0.055
HOMA-IR	1.94 (1.38–2.47)	1.50 (1.20–1.98)	0.079
Total cholesterol, mg/dL	219.6 (187.4–249.8)	173.2 (158.4–187.6)	**<0.001**
LDL-C, mg/dL	132.0 (101.0–156.0)	106.5 (94.0–120.0)	**0.006**
HDL-C, mg/dL	52.0 (42.0–67.0)	46.0 (38.0–52.0)	**0.044**
Triglycerides, mg/dL	133.0 (102.0–189.0)	83.5 (66.0–118.0)	**<0.001**
Adiponectin, ng/mL	15.6 (11.7–48.3)	39.3 (25.9–44.1)	**0.034**

AI, adrenal incidentaloma; FBG, fasting blood glucose; HOMA-IR, homeostasis model assessment of insulin resistance; LDL-C, low-density lipoprotein cholesterol; HDL-C, high-density lipoprotein cholesterol.

**Table 2:** Hormonal profiles and adenoma diameters of the patients with adrenal incidentaloma.

	Patients with AI		*p*
	Patients with SCS	Patients with NAI	
	Median (*Q*1–*Q*3)	Median (*Q*1–*Q*3)	
Adenoma diameter, cm	3.05 (2.25–3.55)	1.85 (1.60–3.00)	0.154
Basal cortisol at 08:00 a.m., *µ*g/dL	16.30 (13.20–24.65)	17.05 (12.40–21.25)	0.554
Midnight cortisol, *µ*g/dL	9.15 (7.00–12.25)	5.10 (3.80–7.80)	**0.004**
ACTH, pg/mL	9.25 (8.60–12.00)	10.70 (8.55–13.00)	0.612
DHEAS, mg/dL	22.10 (15.00–41.95)	51.65 (42.00–113.00)	0.054
UFC, *µ*g/24 h	249 (215–366)	170 (140–220)	**0.007**

AI, adrenal incidentaloma; SCS, subclinical Cushing's syndrome; NAI, nonfunctional adrenal incidentaloma; ACTH, adrenocorticotropic hormone; DHEAS, dehydroepiandrosterone sulfate; UFC, urinary free cortisol.

**Table 3:** Demographic, clinical, and metabolic characteristics of the study groups.

	SCS group (*n* = 8)	NAI group (*n* = 32)	Control group (*n* = 30)	*p*
	*n* (%)	*n* (%)	*n* (%)	
Gender				
Female	5 (62.5)	19 (59.4)	22 (73.3)	0.501
Male	3 (37.5)	13 (40.6)	8 (26.7)	

	Median (*Q*1–*Q*3)	Median (*Q*1–*Q*3)	Median (*Q*1–*Q*3)	

Age, years	56.5 (51.5–64)	61 (56–66)	32 (24–44)	**<0.001**
Body mass index, kg/m^2^	26.8 (25.0–31.5)	29.4 (26.4–31.9)	25.8 (22.8–28.3)	**0.012**
Waist circumference, cm	96 (85.5–100.5)	97.5 (91.5–104.5)	85 (83–90)	**<0.001**
Waist-to-hip ratio	0.91 (0.81–0.98)	0.90 (0.86–0.94)	0.93 (0.92–0.95)	0.138
Body fat percentage, %	27.7 (21.5–35.1)	34.8 (25.8–43.6)	29.1 (26.0–33.0)	0.075
Fat-free mass, kg	49.9 (46.3–56.0)	48.4 (44.5–55.9)	44.8 (41.1–56.8)	0.272
FBG, mg/dL	95 (84–121)	93.5 (88.5–107.5)	91 (88–97)	0.338
Insulin, *μ*IU/mL	13.3 (10.0–15.5)	7.9 (4.5–9.8)	6.6 (4.8–8.1)	**0.009**
HOMA-IR	2.76 (2.07–4.17)	1.73 (1.01–2.08)	1.50 (1.20–1.98)	**0.018**
Total cholesterol, mg/dL	193.2 (179.6–223.8)	221 (187.4–251.6)	173.2 (158.4–187.6)	**0.001**
LDL-C, mg/dL	125.0 (108.0–134.5)	139.0 (101.0–162.0)	106.5 (94.0–120.0)	**0.014**
HDL-C, mg/dL	42.0 (41.5–47.5)	54.0 (44.0–68.0)	46.0 (38.0–52.0)	**0.018**
Triglycerides, mg/dL	135.5 (103.5–241.0)	133.0 (102.0–165.0)	83.50 (66.0–118.0)	**0.002**
Adiponectin, ng/mL	12.0 (7.3–12.8)	24.4 (13.1–48.8)	39.4 (25.9–44.1)	**0.001**

SCS, subclinical Cushing's syndrome; NAI, nonfunctional adrenal incidentaloma; FBG, fasting blood glucose; HOMA-IR, homeostasis model assessment of insulin resistance; LDL-C, low-density lipoprotein cholesterol; HDL-C, high-density lipoprotein cholesterol.

**Table 4:** Relationship of adiponectin with other parameters.

	Adiponectin		
	*n*	rho	*p*
Age	69	–0.077	0.532
Adenoma diameter	39	–0.166	0.312
Body mass index	69	–0.017	0.892
Fasting blood glucose	69	–0.098	0.421
Insulin	58	–0.387	**0.003**
HOMA-IR	58	–0.391	**0.002**
Total cholesterol	65	–0.191	0.127
LDL-C	66	–0.235	0.057
HDL-C	65	0.386	**0.001**
Triglycerides	66	–0.504	**<0.001**
Body fat percentage	69	0.294	**0.014**
Fat-free mass	69	–0.502	**<0.001**
Waist circumference	69	–0.23	0.057
Waist-to-hip ratio	69	–0.159	0.192
Basal cortisol	69	–0.112	0.361
Midnight cortisol	39	–0.359	**0.025**
ACTH	65	0.284	**0.022**
UFC	25	–0.372	0.067
DHEAS	36	–0.402	0.015

HOMA-IR, homeostasis model assessment of insulin resistance; LDL-C, low-density lipoprotein cholesterol; HDL-C, high-density lipoprotein cholesterol; ACTH, adrenocorticotropic hormone; UFC, urinary free cortisol; DHEAS, dehydroepiandrosterone sulfate.

**Table 5:** Risk factors affecting the adiponectin level.

	*B* or coefficients	*p*
Constant	5.748	<0.001
Age	0.012	0.066
Gender	−0.708	0.001
HOMA-IR	−0.229	0.024
LDL-C	−0.005	0.068
Waist circumference	−0.019	0.114
SCS	−1.009	0.005

Adiponectin = 5.748 + 0.012 *∗* age + (−0.229) *∗* HOMA-IR + (−0.005) *∗* LDL-C + (−0,019) *∗* waist circumference + (−1,009) *∗* SCS + (−0,708) *∗* gender (male).

HOMA-IR, homeostasis model assessment of insulin resistance; LDL-C, low-density lipoprotein cholesterol; SCS, subclinical Cushing's syndrome.
